# A Digital Gaming Intervention to Improve HIV Testing for Adolescents and Young Adults: Protocol for Development and a Pilot Randomized Controlled Trial

**DOI:** 10.2196/29792

**Published:** 2021-06-24

**Authors:** Amanda D Castel, Brittany Wilbourn, Connie Trexler, Lawrence D D'Angelo, Daniel Greenberg

**Affiliations:** 1 Department of Epidemiology Milken Institute School of Public Health George Washington University Washington, DC United States; 2 Adolescent Clinical Research Children's National Hospital Washington, DC United States; 3 Division of Adolescent and Young Adult Medicine Children's National Hospital Washington, DC United States; 4 Media Rez Washington, DC United States

**Keywords:** HIV testing, pre-exposure prophylaxis (PrEP), youth, mobile phone

## Abstract

**Background:**

Two strategies of the US Ending the HIV Epidemic initiative are early diagnosis of infections via widespread testing and prevention of new infections using pre-exposure prophylaxis (PrEP). These strategies are particularly important for adolescents and young adults (AYAs) who are disproportionately affected by HIV, particularly if they identify as Black and/or lesbian, gay, bisexual, transgender, queer or questioning, and others (LGBTQ+). This study will develop and test an interactive life-simulation game in which players can enact real-life behaviors and receive their HIV risk profile to improve HIV testing and PrEP access among AYAs aged 13-24 years in Washington, DC.

**Objective:**

This mixed methods study aims to determine the acceptability of an interactive, enhanced life-simulation game prototype among AYAs, conduct a pilot test of the gaming intervention among a small cohort of AYAs to ensure game usability and acceptability, and evaluate the efficacy of the game in a randomized controlled study with AYAs at risk for HIV in Washington, DC.

**Methods:**

This research protocol will be conducted in 3 phases. A formative phase will involve surveys and focus groups (n=64) with AYAs living in the DC area. These focus groups will allow researchers to understand youth preferences for game enhancement. The second phase will consist of a pilot test (n=10) of the gaming intervention. This pilot test will allow researchers to modify the game based on formative results and test the planned recruitment and data collection strategy with intended end users. The third phase will consist of a randomized controlled study among 300 AYAs to examine the efficacy of the life-simulation game compared with app-based HIV educational materials on HIV and PrEP in changing HIV testing, knowledge, risk behaviors, and PrEP access. Participants will have unlimited access to either the life-simulation game or the educational app for 3 months from the time of enrollment. Study assessments will occur at enrollment and at 1, 3, and 6 months post enrollment via e-surveys. At 6 months, a subset of intervention participants (n=25) will participate in in-depth *exit* interviews regarding their experience being in the study.

**Results:**

Institutional review board approval was received on February 5, 2020. This project is currently recruiting participants for the formative phase.

**Conclusions:**

This interactive life-simulation intervention aims to increase HIV testing and PrEP access among AYAs in the DC area. In this intervention, players can enact real-life behaviors and receive their HIV risk profile to promote HIV testing and PrEP seeking. Such an intervention has great potential to improve knowledge of HIV and PrEP among AYAs, increase motivation and self-efficacy related to HIV testing and PrEP use, and decrease individual and structural barriers that often preclude engagement in HIV prevention services.

**Trial Registration:**

ClinicalTrials.gov NCT04917575; https://clinicaltrials.gov/ct2/show/NCT04917575

**International Registered Report Identifier (IRRID):**

PRR1-10.2196/29792

## Introduction

### Background

The goals of the US National HIV AIDS Strategy, Ending the HIV Epidemic initiative, and the HIV care continuum to reduce new HIV infections by intensifying prevention efforts where HIV is most heavily concentrated [1-3] are particularly important for adolescents and young adults (AYAs) aged 13-24 years who are disproportionately impacted by HIV. In 2018, AYAs accounted for 21% of new HIV infections, and young men who have sex with men (YMSM) and transgender women who identified as Black or Latinx were most severely affected [4]. In Washington, DC, a geographic hotspot for HIV [3], AYAs accounted for 21% of new diagnoses in 2019 [5]. Furthermore, Black youth and YMSM in DC accounted for 75% and 60% of new HIV diagnoses among youth in 2019, respectively [6]. Thus, creative strategies to support risk assessment, prevent HIV infection, encourage routine HIV testing, and link AYAs to care are increasingly paramount.

Barriers to HIV testing among youth include difficulty identifying and accessing HIV testing sites, low health literacy and beliefs that HIV remains a severe and fatal disease, and lack of self-perceived risk for HIV [7-12]. Aside from HIV testing, the use of other HIV prevention interventions, such as pre-exposure prophylaxis (PrEP), has been limited among AYAs. Hence, there is a need to improve youth understanding of HIV, how to prevent it, and behaviors that put one at risk for infection and to provide them with resources to access HIV testing and PrEP, in a youth-friendly and nonjudgmental manner [8,13,14]. A review of HIV testing interventions focused on increasing adolescents’ intentions to obtain HIV testing found that facilitators of testing include understanding testing as an essential part of accessing early treatment, helping youth think about future health, and learning how to effectively communicate with their sexual partners [12]. Furthermore, with the US Food and Drug Administration expansion of PrEP to adolescents and the US Preventive Services Task Force Grade A recommendation for PrEP, options for HIV prevention interventions for AYAs have expanded [15-17].

Despite the fact that AYAs are more receptive to HIV information when it is provided in an entertaining format [12,18] and when the material comes from a peer [19-21] or is shared over social media, interventions to promote HIV testing among AYAs have been limited. Digital game–based interventions are a potential strategy to increase HIV testing among AYAs as they overcome traditional barriers faced by this group [22] and can educate while simultaneously motivating AYAs [23]. In addition, paradata, or information related to the process of data collection, can be used to assess the levels of intervention exposure and can assist in further tailoring interventions to individual AYAs. Paradata can potentially inform which elements of HIV interventions are most useful, should be modified, or removed to increase an intervention’s efficacy and can assist in tailoring game-based interventions in a timely and cost-effective manner [24].

### Theoretical Framework for Intervention

We will use social cognitive theory (SCT) and the health belief model (HBM) to ground our intervention development. SCT provides a framework relevant to both health behaviors and interventions and has been widely used in HIV interventional studies [24-27]. We hypothesize that the game intervention will positively influence youth self-efficacy, risk perceptions, and knowledge of HIV prevention as well as influence AYAs’ social support. The HBM proposes that health behaviors are influenced by a person’s perceived susceptibility and seriousness of a disease and the benefits of acting to reduce that risk [28]. HBM has been used to assess HIV testing uptake among various populations, including AYAs [29-32]. We expect that as AYAs navigate and experience the various game scenarios, encounter persons of different HIV risk, and learn about HIV and associated risks, the game will increase HIV risk perception, increase HIV and PrEP knowledge, and improve self-efficacy with respect to sexual relationships and HIV prevention, that is, seeking HIV testing, PrEP, or reducing risky sexual behaviors.

### Aims and Objectives

This mixed methods study will enhance a life-simulation game intervention for AYAs at risk for HIV in the Washington, DC, metropolitan area. The game will be informed by findings from the focus groups with 64 AYAs. We will then conduct a pilot test with 10 AYAs and a randomized controlled trial (RCT) with 300 AYAs. To evaluate the efficacy of the life-simulation game compared with app-based HIV educational materials on HIV and PrEP in changing HIV testing, knowledge, risk behaviors, and PrEP access, RCT participants will have unlimited access to either the game or the educational app for 3 months from the time of enrollment. Electronic study assessments will occur at enrollment and at 1, 3, and 6 months post enrollment. At 6 months, a subset of intervention participants (n=25) will participate in in-depth *exit* interviews regarding their experience being in the study.

## Methods

### Participants

AYAs will be eligible for enrollment in each phase of the study based on the following criteria: (1) ages 13-24 years; (2) English speaking; (3) sexually active; (4) HIV-negative status; (5) residing in DC, Maryland, or Virginia; and (6) able to provide informed consent or assent. AYAs participating in the second and third phases will also need to have a mobile phone that they are willing to use for the study. We are limiting the study to AYAs aged 13-24 years because they account for a significant proportion of new HIV cases in Washington, DC, and doing so will allow for the development of a life-simulation gaming intervention that is targeted, acceptable, and engaging for this specific population. We plan to enroll a sample representative of diverse racial and ethnic and sexual behaviors.

### Description of Intervention Content

**Figure 1:**
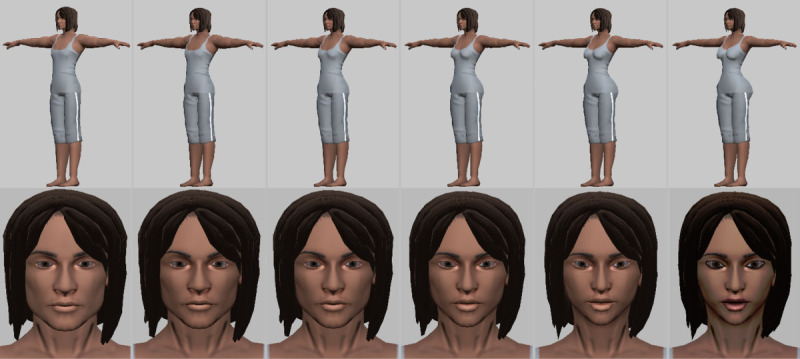
Draft avatar editor system showing a sample character that can transition from masculine to feminine gender expression.

**Figure 2:**
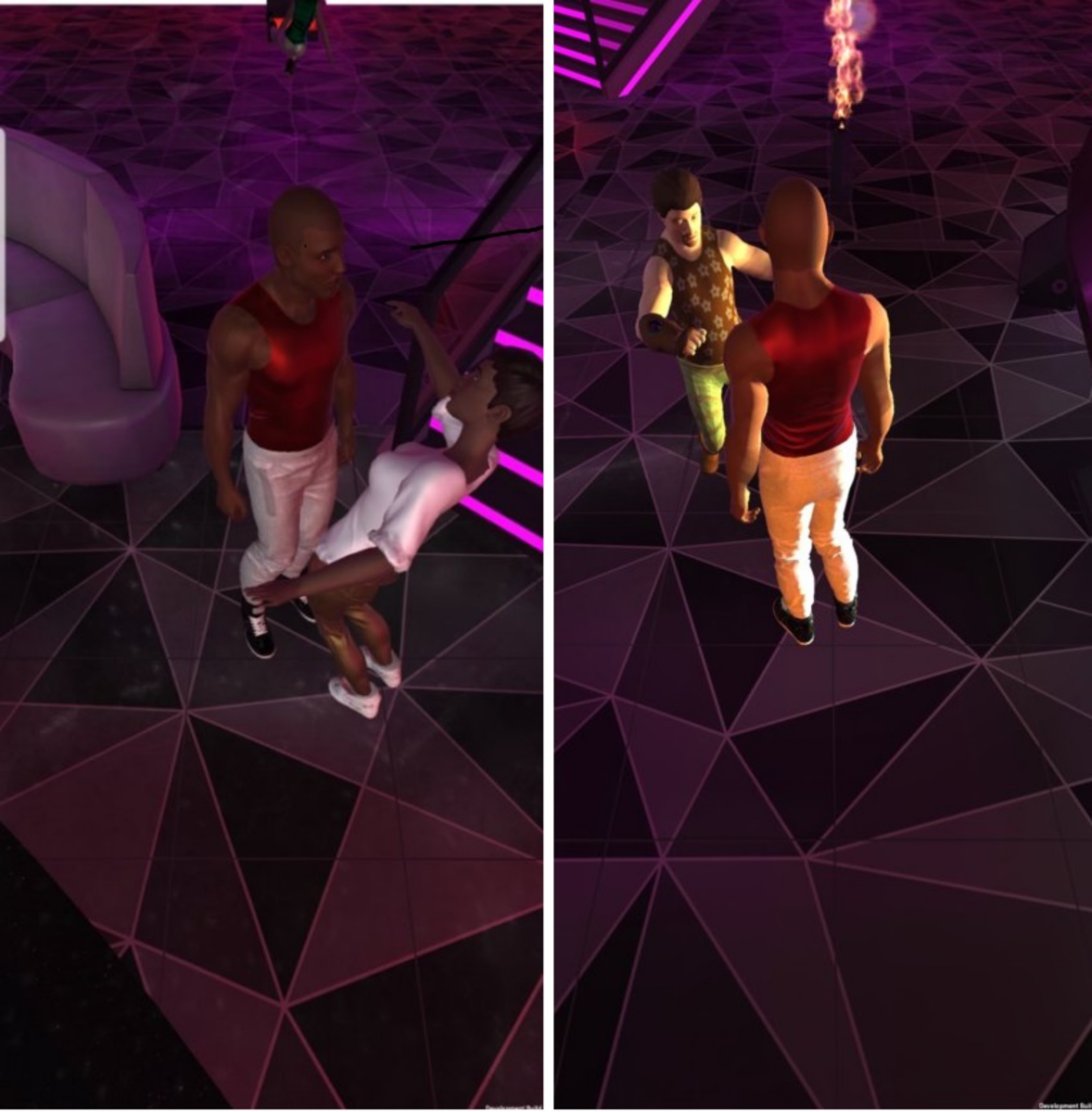
Draft scene of opposite sex and same sex flirtation.

The life-simulation game prototype for smartphones (iOS and Android), tablets, and the web was developed in a phase 1 Small Business Innovation Research study [33] and shows an AYAs’ HIV risk based on their sexual choices in the game. Several areas for modification were identified in the phase 1 study, including the incorporation of a customizable avatar; the development of additional activities, locations, and dating scenarios; incorporation of data analytics features to collect game and paradata; and access to a PrEP provider locator. The incorporation of these modifications has resulted in an enhanced life-simulation game that will be further refined throughout the course of the study (Figure 1). Players can create their in-game *self* using a system that unifies all forms of racial and gender expression into a single avatar, allowing both cisgender and unique transgender and nonbinary characters customized to the player’s specifications. Our previous data suggest that increasing in-game self-expression can result in player identification with their onscreen character, concern for their character’s HIV risk profile, and interest in HIV testing and prevention. The player’s character can seek to date same-sex partners, opposite-sex partners, both, or neither (Figure 2). The game features in-depth dialog for players to explore social interactions for both dating and platonic relationships. Fresh branches of storylines are opened up or shut down based on the player’s choices. The game directly links both rewards and penalties to the player’s choices, which establishes credibility with players that the game is not arbitrary but displays real-life consequences for all their actions. This credibility is necessary for the consequences of their sexual choices to be meaningful and impactful. When the player’s flirtations succeed, a screen appears with their chosen partner and a range of sexual choices based on the sex of the player and their partner [33]. For each sexual act selected, the player is prompted to decide whether to use condoms. When the player clicks *Let’s Get It On*, the player hears romantic music and fireworks burst onto the screen to imply sexual activity, with no graphic content displayed.

**Figure 3:**
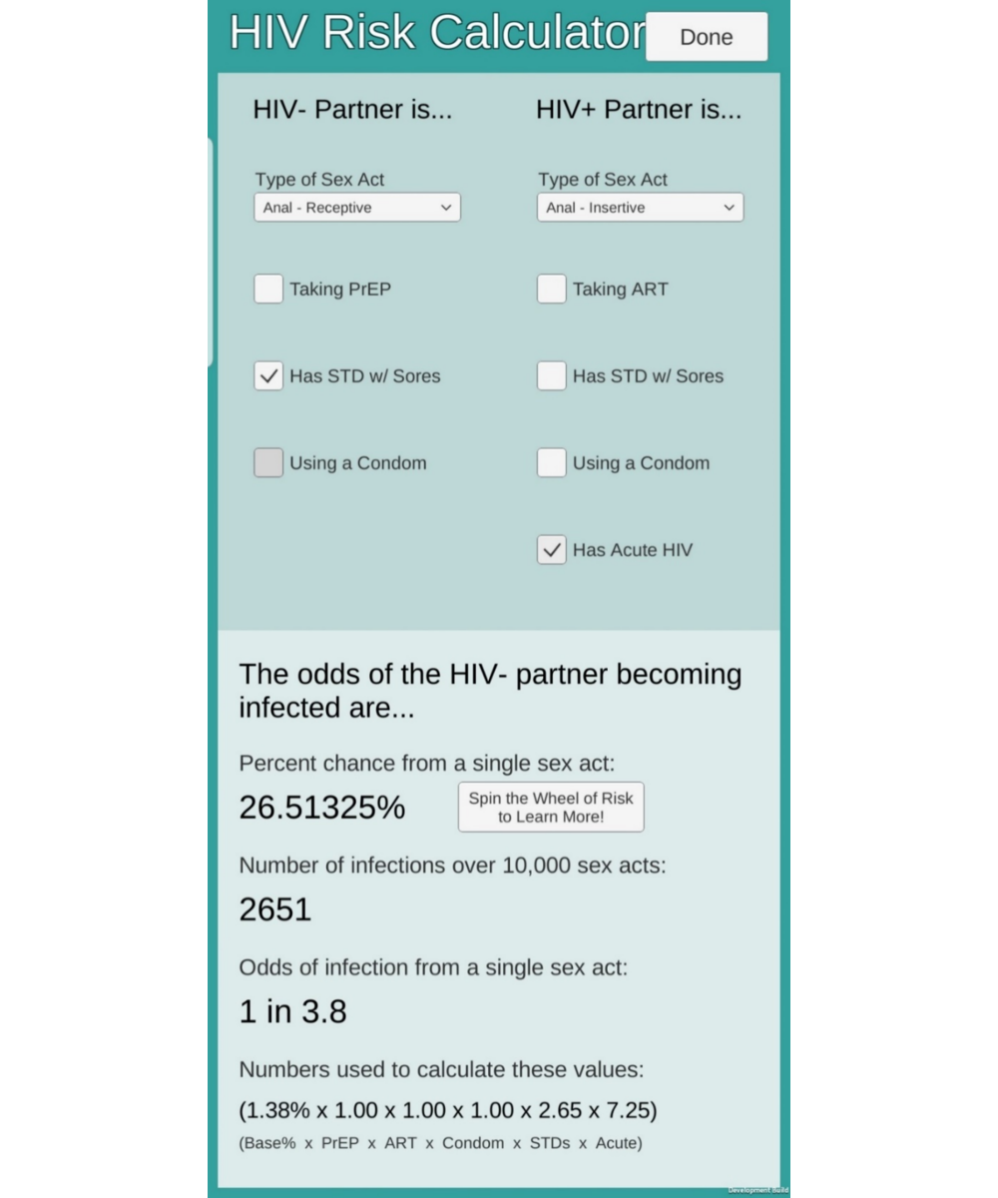
Draft HIV risk screen adapted from the Centers for Disease Control and Prevention risk estimator tool.

**Figure 4:**
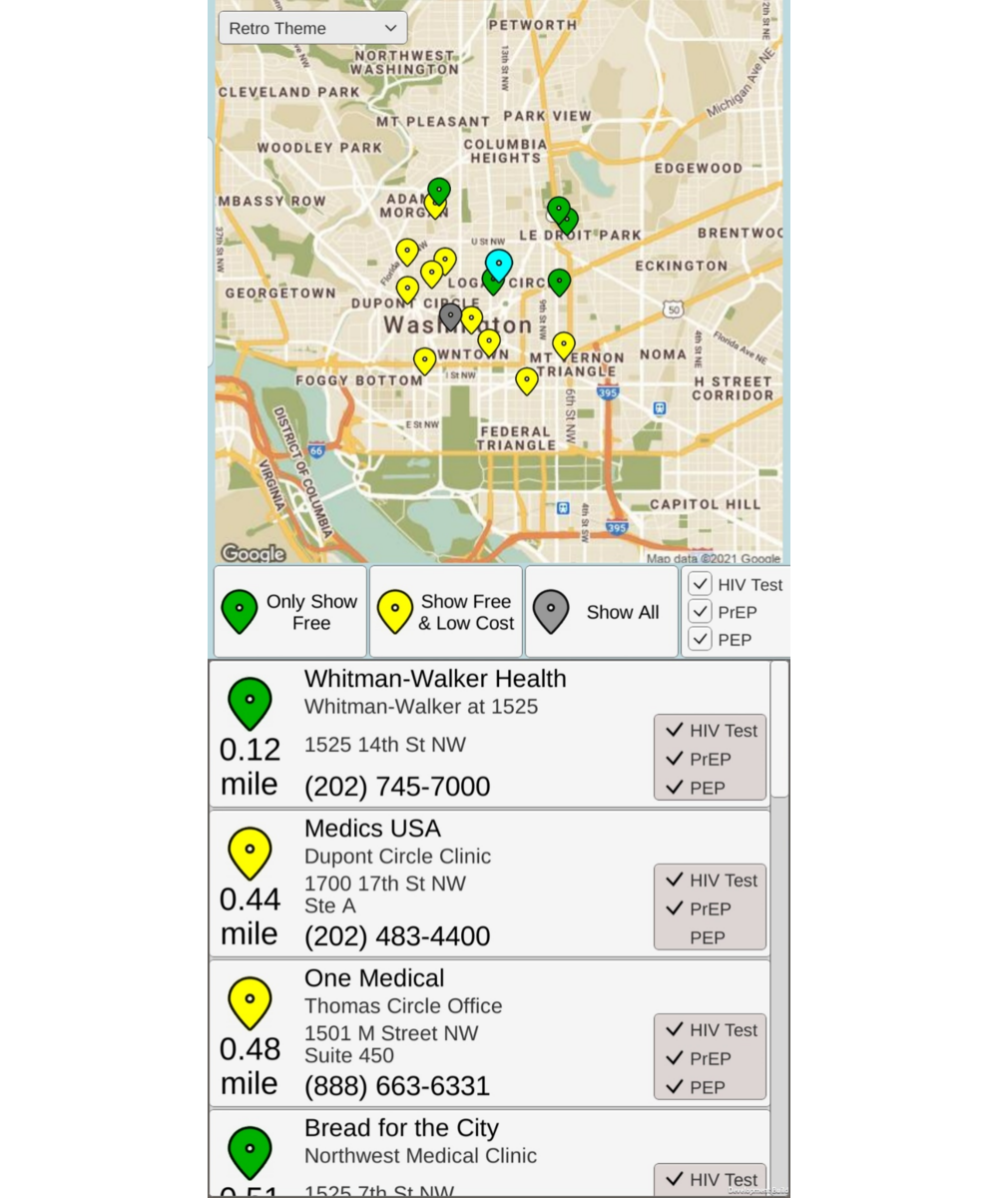
HIV prevention services locator based on publicly available the Centers for Disease Control and Prevention locators.

After the implied sexual activity, the music abruptly stops, and the player sees the estimated HIV risk their character just incurred (Figure 3). The player is then offered an opportunity to locate low-cost or free HIV testing sites near them by inputting their ZIP code or giving the game permission to read their phone’s location. Data from the US Centers for Disease Control and Prevention testing locator appear with details on where to get tested locally (Figure 4). Although AYAs do not see their HIV risk immediately after a real-life sexual encounter, AYAs in this game are directly confronted by the reality of the risk their character incurs. Our phase 2 study will test whether this system helps reinforce the connection between sex and risk, thereby improving HIV testing.

### Formative Focus Groups

Focus groups will occur in 2 rounds (n=32 each) and will be conducted by ADC, DG, and BW, who have experience with qualitative research used to develop behavioral interventions. Focus group participants will be recruited via advertisements placed on various social media sites (eg, Facebook, Snapchat, and Instagram). Interested participants will be directed to a web-based eligibility screener in the REDCap (Research Electronic Data Capture) platform, a secure Health Insurance Portability and Accountability Act (HIPAA)–compliant web application designed exclusively to support data capture for research studies [34], to determine their age, gender, sexual behaviors and orientation, and state of residence. Eligibility will also be confirmed by the project staff via phone. Those eligible and interested will be sent a link to survey in REDCap, with the consent and assent forms embedded, meant to collect information on youth sociodemographics, use of games and social media, knowledge of HIV and PrEP, sexual risk behaviors, and experiences with HIV testing and PrEP use. Eligible and interested participants will also provide information on the date and time of the focus. Focus groups will be stratified by sexual orientation (heterosexual vs lesbian, gay, bisexual, transgender, queer or questioning, and others [LGBTQ+]), and age (13-17 years vs 18-24 years). Before the focus groups, the research staff will review the consent and assent forms with participants and obtain verbal consent and assent before proceeding with the focus group.

Focus group questions cover the following topics: HIV and PrEP knowledge, perceptions and experiences regarding HIV testing, current use of and access to gaming technology (eg, use of smartphones, game consoles), perceptions regarding game enhancements and their potential role in increasing HIV testing and access to PrEP, perceptions about the role of social media and its influence on HIV testing behaviors, and opinions regarding effective recruitment approaches through social media. The enhanced life-simulation game will be demonstrated to participants, and feedback will be elicited regarding the acceptability and relevance of characters, texts, actions, and graphics, as they relate to race, culture, and structural factors. Following the focus group, participants will be sent the link to a survey in REDCap to assess their change in knowledge of HIV and PrEP as well as their satisfaction with the game prototype.

All focus groups will be audio recorded, and a verbatim transcription is professionally produced. The resulting text file will be entered into the qualitative data analysis software Atlas.ti 7.0 (ATLAS.ti Scientific Software Development GmbH) and analyzed using an a priori and open coding process aimed at identifying relevant themes and categories to inform the development of the game modifications for round 2 focus group testing as well as the final game prototype in the pilot test. Key decisions will include potential differences by age and sexual orientation, the types of games, art styles, social features, type or frequency of in-game bonuses, and responses of different kinds of in-game virtual rewards for actions such as demonstrating knowledge of HIV risks and virally spreading the game to peers. Game modifications will be made based on the initial focus group feedback (n=32), after which the second group of participants (n=32) will be recruited and shown the revised game. Survey data will be cleaned, downloaded, and exported into Excel or SAS files, and descriptive statistics will be analyzed. Pre- and postgame knowledge levels will be calculated; however, because of the small sample size, no statistical testing will be performed.

### Pilot Test

#### Finalization of Game Prototype

If it is determined that AYA in the formative focus group does not like the enhanced life-simulation game, additional modifications will be made. Once the look and feel have been finalized, the game production team will implement v2.0, creating base code, audio, and 3D art or animation using the Unity *middleware* engine.

#### User Field Testing

Pilot test participants will be recruited via advertisements placed on various social media sites. Interested participants will be directed to a web-based eligibility screener in REDCap. Participant eligibility will be confirmed by the project staff via phone. Eligible participants will provide electronic consent and assent and complete the baseline survey electronically via a HIPAA-compliant participant management database. Participants will then be sent the gaming app electronically to download on their phone and will have unlimited access for 1 month. User pilot testing will allow project staff to identify any unanticipated issues with game log-ins, navigation, functionality, paradata collection, or other unforeseen technical issues. During user testing, game use as well as HIV testing and PrEP locator use will be monitored and paradata will be accessed for a 1-month period.

We will also pilot the use of the HIPAA-compliant participant management database for participant screening, survey collection, and data management and troubleshoot for any issues with phone connectivity, data caching, paradata collection, or other potential issues. Baseline data on HIV testing and knowledge will be collected to preliminarily describe the anticipated characteristics of our web-based sample. Measures of game satisfaction [35], usability [36], and a modified version of the motivation, attitude, knowledge, and engagement instrument [37] will also be piloted to determine how well the game addresses HIV knowledge and testing. At the end of the 1-month period, brief surveys and in-depth interviews (IDIs) will be conducted with the 10 pilot participants to elicit features of the games that worked well and identify those that might prohibit full use of the intervention.

Survey data will be cleaned, downloaded, and exported into Excel or SAS files. Given the small sample size and objectives of this study, quantitative analysis will be primarily descriptive to measure usability, acceptability, and satisfaction as well as to confirm baseline characteristics and behaviors of our intended study population. All interviews will be audio recorded, and verbatim transcription professionally produced. The resulting text file will be entered into the qualitative data analysis software Atlas.ti 7.0 and analyzed using an a priori and open coding process aimed at identifying potential barriers and facilitators to use. Critical and essential modifications will be made to the game, paradata, and HIPAA-compliant data management platforms before the RCT.

### Randomized Controlled Trial

#### Overview

**Figure 5:**
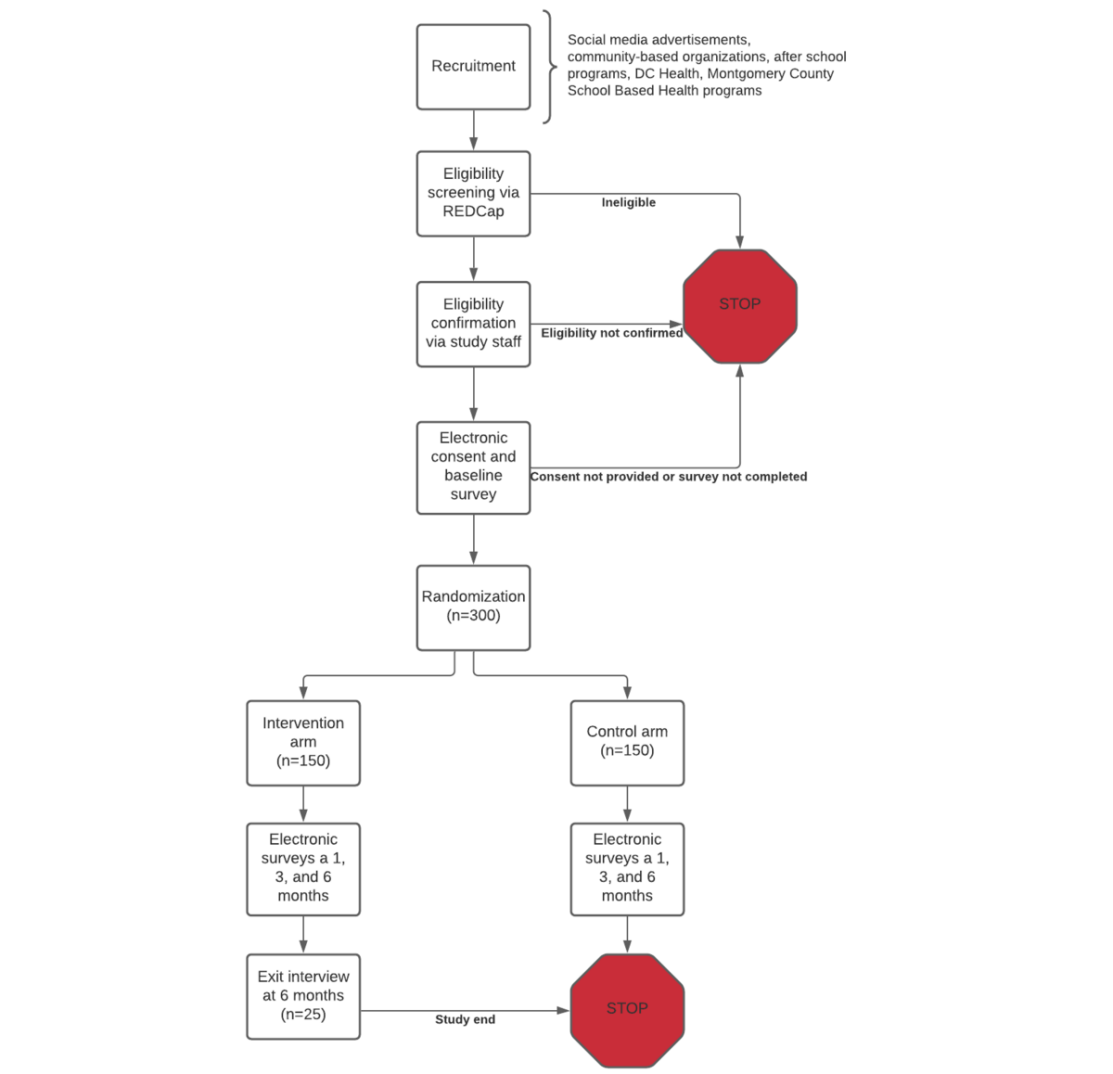
Aim 3 study flow diagram. DC: District of Columbia; REDCap: Research Electronic Data Capture.

We will evaluate the impact of the life-simulation game compared with app-based HIV educational materials in an RCT with 300 AYAs. To enroll a sample representative of diverse racial or ethnic and sexual behaviors as well as a sample of non–clinic-attending AYAs, recruitment will occur primarily through social media. We will place targeted advertisements toward YMSM and Black and Hispanic AYAs on social media sites, such as Facebook, Instagram, and Snapchat. In addition, we will provide recruitment materials with quick response codes to the DC Health and Montgomery county school-based health programs. Interested AYAs will click on an advertisement and be directed to a web-based eligibility screener to determine their age, gender, sexual behaviors and orientation, state of residence, and availability of a reliable cell phone. The eligibility of the participants will be confirmed by the project staff via phone. Eligible participants will then provide electronic consent and complete the baseline survey electronically. Participants will be randomized using the block randomization method designed to ensure groups of equal sample sizes (1:1). Participants will be randomized in blocks of 6, stratified by age (13-17 years and 18-24 years) and sexual behavior (LGBTQ+ vs heterosexual), using a random number generator for assignment. Randomization will occur through the HIPAA-compliant participant management database platform. We will compare conditions on HIV testing, knowledge, risk behaviors, and PrEP access at enrollment and at 1, 3, and 6 months post enrollment via electronic survey assessments. At 6 months, a subset of intervention participants (n=25) will participate in in-depth *exit* interviews regarding their experience being in the study. A schematic of the study design is shown in Figure 5.

#### Intervention Delivery

Intervention participants will have access to the full revised study game for 3 months from the time of enrollment and will be able to play the game and have up to 30 hours of content available. To sustain interest and engagement over the study period and maximize potential efficacy, new downloadable game content will be available every 4 weeks post enrollment through the 3-month follow-up.

#### Control Condition

Before rolling out the RCT, we will develop an app that will include basic information on HIV (eg, routes of transmission, data on the epidemiology of HIV among youth, prevention information, and information on HIV testing and PrEP) as well as a link to the HIV risk estimator and HIV testing and PrEP locators. Participants assigned to the control arm will receive a link to download this informational app containing this content and will have unlimited access to the app for a 3-month period.

#### Measures

Electronic surveys via REDCap will be used to assess the behavior at baseline and at 1, 3, and 6 months post enrollment, as it is confidential and allows for complex branching or skip patterns [34]. Participants will be emailed the REDCap surveys 7 days before their designated completion date, on their completion date, and 7 days after their completion date if not completed. Standard items will be administered to gather demographic data (including age, educational level, sexual orientation, socioeconomic status, race, and ethnicity). Surveys will collect information on the following validated domains: HIV- and PrEP-related knowledge; self-reported sexual and HIV risk behaviors; self-perceived risk for HIV; perceived norms; barriers to HIV testing; reported HIV testing and PrEP uptake; behavioral intentions including intent to test for HIV; seeking PrEP; and reducing risky behaviors, self-efficacy, and game use, interest, acceptability, and usability.

At 6 months, a purposely selected subset of intervention participants (n=25) will participate in IDIs regarding their experience being in the study. The IDIs will be conducted by trained qualitative interviewers and will follow semistructured interview guides. The IDIs will assess the extent to which app components facilitate or inhibit HIV testing. Central to the IDIs will be understanding the participants’ use of the app. To obtain a range of perspectives, we will seek feedback from participants stratified by sexual behaviors (heterosexual vs LGBTQ+), age group (13-17 years vs 18-24 years), whether they sought and received HIV testing, and levels of game play and locator use based on the paradata (none, low-moderate, and high use).

### Outcomes

#### Primary Outcomes

The primary outcome measure for the study is HIV testing. This will be measured as self-reported HIV testing in the past 6 months. For all participants who report testing, we will attempt to validate reported testing by eliciting a description of the HIV testing process and requesting that a secure photo of their test results be sent to study staff to minimize social desirability bias. We will compare self-reported HIV testing between the 2 groups at 6 months.

#### Secondary Outcomes

As self-reported measures tend to overestimate HIV testing and given the age range of our participants and potential structural barriers to the receipt of HIV testing among AYAs (eg, parental knowledge of testing, lack of transportation, inconvenient clinic times), a secondary outcome will be intent to test for HIV. The intent to test will be assessed using a validated 6-point Likert scale [38], shown to be predictive of future testing behavior, with responses ranging from *very unlikely* to *extremely likely* to test in response to the question. “how likely are you to get tested in the next 6 months?” We will dichotomize this score into *extremely likely* versus other. Other secondary outcomes will use previously published questions and validated scales (Table 1) and include changes in (1) HIV knowledge [39]; (2) risk perception [40] and behaviors (eg, condomless sex); (3) PrEP knowledge [41,42], screening [41,42], and uptake (also verified with a photograph of a pill bottle or prescription); (4) risk estimator scores and locator (HIV and PrEP) use; and (5) game acceptability [35] and usability [36], including frequency and duration of use, and reasons for continued or discontinued use.

**Table 1:** Measures planned for each phase.

Domain			Assessment time		
			Focus group	Pilot test	RCT^a^
**Primary outcome**					
	**HIV testing**				
		Tested during intervention period^b^	✓^c^	✓	✓
		Interval and frequency of test	✓	✓	✓
		Reason for test [43]	✓	✓	✓
		Perceived benefit of HIV testing	✓	✓	✓
**Secondary outcomes**					
	**Behavioral intentions**				✓
		Intent to test [38]			✓
		Intent to seek PrEP^d^ [41]	✓		✓
		Intent to reduce risky behaviors [44]			✓
	HIV knowledge [39]		✓	✓	✓
	Perceived susceptibility to HIV [40]				✓
	**Risk assessment**				
		Sexual behaviors	✓	✓	✓
		Ever STD^e^ diagnoses	✓	✓	✓
		Alcohol and illicit drug use [45]	✓	✓	✓
	Perceived norms [46]				✓
	**PrEP**				✓
		Knowledge and screening [41,42]	✓		✓
		Use or uptake			✓
	**Self-efficacy or likelihood of action**				
		Ability to negotiate condom use [29]	✓		✓
		Encourage partners or friends or peers to get HIV testing	✓		✓
	**Game and paradata**				
		Game downloads		✓	✓
		Frequency and duration of game play		✓	✓
		Game satisfaction [35]		✓	✓
		Usability [36]		✓	✓
		Teaching approach [37]		✓	✓
		CDC^f^ Risk Estimator scores		✓	✓
		Number of linkages to HIV testing locator		✓	✓
		Number of linkages to PrEP locator		✓	✓
		Recommend app to friend or share on social media		✓	✓
**Modifying factors**					
	Demographics		✓	✓	✓
	Use of social media and gaming		✓	✓	✓
	Exposure to health or sex education programs		✓	✓	✓
	Perceived barriers to HIV testing		✓		✓

^a^RCT: randomized controlled trial.

^b^Verification of HIV testing and pre-exposure prophylaxis uptake will be conducted through photos and in-depth interviews.

^c^The tick mark indicates that the measure will be assessed during that specific phase of the study.

^d^PrEP: pre-exposure prophylaxis.

^e^STD: sexually transmitted disease.

^f^CDC: Centers for Disease Control and Prevention.

### Statistical Analysis

In our pilot study [33], we found that approximately 10% of sexually active AYAs reported being tested for HIV in the past 4-6 months. A sample of approximately 300 subjects (150 per arm) would achieve >80% power to detect a moderate effect corresponding to a difference of at least 13 percentage points (equivalent to a relative risk of 2.3 assuming a 10% prevalence of HIV testing in the past 4-6 months in the control group) in HIV testing between the intervention and control groups after 6 months using a two-sided test at a 5% significance level and assuming a 16% attrition rate after 6 months. All data will be inspected for inaccurate input (eg, out-of-range values, implausible means, and variation). We will first summarize the outcome measures descriptively overall and within each intervention arm at each time point. We will make every effort to obtain all data. Any patterns of missingness will be investigated and reported. Sensitivity analyses, including rerunning analyses with imputed values for missing data, will be conducted to assess the impact of missingness on the study findings.

All primary analyses will be conducted using an intent-to-treat approach. The primary analysis will focus on differences between the treatment and control groups at the 6-month follow-up visit using a chi-square test for independence (the outcome will be taken as having had an affirmative response on the outcome measure during the 6-month study period). We will also investigate the differences between the rates of HIV testing in the treatment and control groups, stratifying by age (13-17 years vs 18-24 years) and sexual orientation (LGBTQ+ vs heterosexual identity). We will perform a longitudinal analysis on the repeated measures from each subject using logistic mixed effects models with a subject-specific random intercept. In each model, we will include time as a 4-level categorical predictor (with baseline serving as the reference level) as well as a treatment indicator and time-by-treatment interaction. We will jointly test the interaction coefficients to determine if the effect of treatment varies over time. If the effect is found to vary over time, we will test the intervention effect at each time point and characterize the difference between the treatment and control groups using risk differences and their corresponding 95% CIs. If no evidence of a time-by-treatment interaction is present, we will refit the logistic model without the interaction terms and provide the overall intervention effect and its corresponding 95% CI.

Furthermore, we will provide a descriptive analysis of each secondary outcome overall and within each intervention arm at each time point. Secondary outcomes will also be compared between the treatment and control groups using one-tailed *t* tests for continuous measures and chi-square tests for categorical measures. The corresponding group differences (in mean scores or proportions) and their 95% CIs will be reported. Within the intervention group, we will investigate game use changes over time and whether there exists a dose-response relationship between various game use indicators (eg, amount of time playing the game) and the various survey responses, including HIV testing, intent to test for HIV, PrEP uptake, and intent to seek PrEP as well as the other secondary outcomes. Qualitative data from IDIs will be transcribed and involve thematic coding using Atlas.ti version 7.0 to identify themes regarding intervention delivery and acceptability.

### Incentives

For their participation in the formative focus groups, AYAs will receive a US $30 gift card and an additional US $10 gift card for a peer recruited who participates in the focus group. For their participation in the pilot test, AYAs will receive US $80 gift cards (US $20 for the baseline survey, US $30 for the 1-month survey, and US $30 for the IDI). For their participation in the RCT, AYAs will receive up to US $150 in gift cards (US $30 for the baseline survey, US $25 for the 1-month survey, US $25 for the 3-month survey, US$40 for the 6-month surveys, and US $30 for the IDI).

### Ethical Considerations

To protect participants from potential loss of confidentiality, the following measures will be taken: informed consent and assent will be required for participation in the focus groups, pilot testing, and intervention. Each study participant will be assigned a pseudonym to participate in the focus groups, and interviews and surveys will be labeled with a study ID. Participants will be informed that individual results will not be shared, and only aggregate results will be disclosed at study completion. Summary statistical data stratified by age, race, or other categories may be released. However, if the total information will allow identification of the exact person or persons the information will not be released.

All study-related materials will only be accessible to the research staff. All data collection will take place with HIPAA-compliant software (ie, WebEx and REDCap). All study personnel have completed training and received certification in Human Subjects Research Protection (Collaborative Institutional Training Initiative Program) and HIPAA regulations and will continue to renew this training in compliance with institutional review board policies. We will adapt measures successful in other studies to reduce the risk of potential breaches by using passwords, not sending any sensitive information, and eliciting AYAs’ preferred method of communication and receipt of study information (eg, surveys and incentives).

## Results

The study was funded on April 2, 2020. The protocol has been reviewed and approved by the George Washington Institutional Review Board (NCR191708) on February 5, 2020 and was registered on ClinicalTrials.gov (NCT04917575). Data collection for the formative phase is projected to begin in June 2021.

## Discussion

### Review

The life-simulation gaming intervention aims to increase HIV testing and PrEP access among AYAs by reducing individual and structural barriers. Through digital content aligned with SCT and the HBM, the intervention attempts to positively influence youth self-efficacy, risk perceptions, and knowledge of HIV prevention as well as influence AYAs’ social support.

### Limitations and Anticipated Challenges

There are several potential challenges to the success of our trial. First, if it is determined that AYAs in the formative focus groups do not like the proposed enhancements, we will modify the game accordingly. Second, for all 3 phases, we plan to intentionally recruit AYAs who are not regular clinic attendees and rely primarily on social media recruitment. If this proves challenging, we will consider expanding recruitment to local community-based organizations that are not HIV focused and consider recruiting from after-school programs or events geared toward AYAs. Third, although web-based recruitment is ideal to identify our population of interest, there is a risk of fraud, and we plan to have staff verify the eligibility of potential participants. Finally, AYAs’ gaming preferences change far faster than the pace of research. Avatar clothes and other in-game items may no longer be popular during the game’s release. The software architecture is designed to be modular and flexible to allow many elements to be altered both before and after release. To ensure adequate game content to keep AYAs engaged during the study period, as per industry standards, we will build in frequent releases of new game content.

### Conclusions

As the number of new HIV infections among AYA continues to grow, innovative methods to scale up HIV prevention for this population are required. The results of this study will be useful for understanding the extent to which a digital game can increase HIV testing and PrEP access among AYAs. If effective, we will work with the local health departments, health clinics, and school-based clinics to make the game available to their students and patients. We will also look into making the game and locators relevant and available to AYAs living in areas of the United States with some of the highest rates of HIV infection, such as the Southern United States.

## Appendix

Multimedia Appendix 1 Peer-review report by the Center for Scientific Review Special Emphasis Panel, Small Business: Disease Prevention and Management, Risk Reduction and Health Behavior Change (National Institutes of Health).

## Abbreviations

AYA: adolescents and young adults
HBM: health belief model
HIPAA: Health Insurance Portability and Accountability Act
IDI: in-depth interview
LGBTQ+: lesbian, gay, bisexual, transgender, queer or questioning, and others
PrEP: pre-exposure prophylaxis
RCT: randomized controlled trial
REDCap: Research Electronic Data Capture
SCT: social cognitive theory
YMSM: young men who have sex with men
