# Supplementary material for: A Digital Gaming Intervention to Improve HIV Testing for Adolescents and Young Adults: Protocol for Development and a Pilot Randomized Controlled Trial
Source: JMIR Res Protoc. 2021 Jun 24;10(6):e29792. doi: 10.2196/29792 (PMC8277397; doi:10.2196/29792)
Supplement: Multimedia Appendix 1 [file resprot_v10i6e29792_app1.pdf]

SUMMARY STATEMENT

(Privileged Communication)

PROGRAM CONTACT:
Sonia Lee
(301) 594-4783
leesonia@mail.nih.gov

Release Date: **07/10/2019**
Revised Date:

Application Number: **2 R44 HD088332-02A1**

Principal Investigators (Listed Alphabetically):

CASTEL, AMANDA DERRYCK
GREENBERG, DANIEL J (Contact)

Applicant Organization: MEDIA REZ, LLC

Review Group: **ZRG1 RPHB-Z (10)**
Center for Scientific Review Special Emphasis Panel
Small Business: Disease Prevention and Management, Risk Reduction and Health Behavior Change
AIDS - EXP. REV.

Meeting Date: **06/24/2019**
Council: **AUG 2019**
Requested Start: **09/01/2019**
RFA/PA: **PA18-573**
PCC: **MPIDB-SL**
Dual IC(s): **MH**

Project Title: **Efficacy of a Dating-and-Life Simulation Game on HIV Testing Uptake among Adolescents and Young Adults**
SRG Action: **Impact Score:23**
Next Steps: **Visit https://grants.nih.gov/grants/next_steps.htm**
Human Subjects: **30-Human subjects involved - Certified, no SRG concerns**
Animal Subjects: **10-No live vertebrate animals involved for competing appl.**
Gender: **1A-Both genders, scientifically acceptable**
Minority: **1A-Minorities and non-minorities, scientifically acceptable**
Age: **2A-Only Children, scientifically acceptable**

Project Year	Direct Costs Requested	Estimated Total Cost
2	302,113	493,918
3	316,272	517,067
4	239,192	391,050
TOTAL	857,577	1,402,035

ADMINISTRATIVE BUDGET NOTE: The budget shown is the requested budget and has not been adjusted to reflect any recommendations made by reviewers. If an award is planned, the costs will be calculated by Institute grants management staff based on the recommendations outlined below in the COMMITTEE BUDGET RECOMMENDATIONS section.

2R44HD088332-02A1 Greenberg, Daniel

RESUME AND SUMMARY OF DISCUSSION: This resubmitted Phase II SBIR uses social media and game technology to increase HIV testing among adolescents and young adults. This age group accounts for 21 percent of new HIV infections but half of those are unaware they are infected. Less attuned to adult-oriented public health interventions, they can be reached more effectively through gameplay. The research problem is serious. Rigor of prior research is assessed. A conceptual model and preliminary data are presented. The investigators were responsive to prior critiques. Reviewers were enthusiastic and accorded the project high significance. The investigative team is experienced and brings the needed interdisciplinary skills. Media Rez, George Washington University and National Children's Hospital afford an ideal research environment. A life simulation game that also exploits social media and seeks to reduce sexual risk-taking by changing attitudes and linking users to care is highly innovative. An iterative, user-center design process to build out an existing prototype game is well described as are quality assurance, usability and efficacy testing methods. The randomized study design, power calculations use of mixed methods and clear data analytic plan enhance scientific rigor. In discussion, several minor weaknesses surfaced. It is unclear which team members bring the needed behavioral content expertise. Qualitative outcomes are not fully described. Whether users will tolerate pop-up ads is questionable. Generalizability beyond Washington, DC is unaddressed. On balance, strengths outweighed weaknesses. The project will significantly advance the prevention of HIV, concluded the panel.

DESCRIPTION (provided by applicant): This study aims to assess the acceptability, usability and efficacy of using state-of-the-art social media and game technology to increase HIV testing among adolescents and young adults (AYA) ages 13-24. Rates of HIV testing among AYA are low, resulting in high proportions of AYA who are positive or unaware of their HIV infection. Further, public health messages which can be effective in older adults do not achieve the same results in AYA. Since many AYA are highly engaged with social media and games, these technologies may hold the key to reaching this population. While previous behavior change games have shown positive results by using the compelling nature of gameplay to make health education entertaining and shift attitudes towards disease prevention, this intervention proposes to incorporate a new innovation which changes the way video games are used to increase HIV testing and linkage to care. The intended result is a novel social media experience which plays like a game while prompting dialogue about HIV risk assessment, testing, and linkage to prevention in a manner that may be more acceptable to AYA compared to traditional public health messages. We have developed an AYA-informed game in which players can create characters like themselves and model different forms of dating and sex practices to learn the consequences in a simulated, non-health or life-threatening environment. Once the players learn the HIV-related risks they have taken in the game, they can identify nearby facilities for HIV testing and prevention services. We seek to test the efficacy of this game and hypothesize that its use will result in increased HIV testing and lead to a reduction in risky behaviors among AYA at risk for HIV. In this Phase II application, our multidisciplinary team will recruit AYA to assist in determining the acceptability of additional game enhancements to the existing prototype through an iterative focus group process. Once an acceptable prototype is develop, we will conduct pilot field testing of the game intervention among a small cohort of AYA to ensure usability, acceptability and to conduct quality assurance testing. Finally, we will conduct a randomized controlled trial to determine the efficacy of the intervention on HIV testing uptake. We will enroll 300 sexually active HIV negative AYA and randomize them to receive either the life-simulation game intervention or provision of an app which contains HIV educational materials on HIV and pre-exposure prophylaxis. We will compare self-reported HIV testing between the two study groups at 6 months and hypothesize that a higher proportion of AYA in the intervention versus control group will have obtained HIV testing. Successful completion of these aims will demonstrate how this product may advance the effective implementation of an evidence-based behavioral intervention for increased HIV testing and linkage to preventive care among youth.

Successful development of this core technology could also be revised to be applicable to other medical conditions.

PUBLIC HEALTH RELEVANCE: This project seeks to determine the efficacy of using a life-simulation game to increase HIV testing, knowledge, risk reduction behaviors, and access to prevention services among young people at risk for HIV. The project focuses on testing an innovative, culturally-appropriate, user- friendly and acceptable youth-appropriate game to help players assess their personal HIV risk through engaging, personalized gameplay. The game rewards players for HIV testing and linkage to care, and sharing the game with others via social networks.

CRITIQUE 1

Significance: 2
Investigator(s): 2
Innovation: 1
Approach: 3
Environment: 1

Overall Impact: This Phase II SBIR resubmission aims to assess the acceptability, usability, and efficacy of using game technology to increase HIV testing among adolescents and young adults (AYA) ages 13-24. Three Aims are proposed: Aim 1 (n=64) will determine acceptability of the interactive game after modifying the existing Phase I prototype to incorporate additional locations, real-life scenarios, and pre-exposure prophylaxis (PrEP) provider locations; Aim 2 will conduct pilot field testing to determine usability (n=10); and Aim 3 will determine the efficacy of the game compared to an educational app in changing HIV testing, knowledge, and risk behaviors among AYA at risk for HIV (n=300) in a randomized controlled trial. In-depth interviews (n=25) will be conducted among users of different levels of exposure to the game (e.g. low, moderate, high) to identify barriers and facilitators of game use. Overall, this resubmission is highly responsive to previous reviews and enthusiasm for the project is high. The study is innovative and the research team is excellent. Scientific premise is well established and rigor via the study design is reasonable and accomplishable. There is commercialization potential for the product. Overall, the identified weaknesses are addressable and are heavily outweighed by the study's strengths.

1. Significance:

Strengths

- HIV remains a significant public health concern especially among AYAs. AYAs account for 21% of new HIV infections. Half of AYAs with HIV infection are unaware of their health status; there are critical gaps in getting at-risk youths tested.
- There is a need to educate this population on HIV risk factors and the value of getting tested in a youth-friendly, nonjudgmental manner. AYAs have high acceptance of testing and HIV information when it is presented in an engaging format.
- Technological formats to educate on HIV are promising, but only a limited number offer gaming components.
- Overall, the Significance is thoughtfully written and supports scientific premise for the application.
- The Commercialization Plan is well-written and logically presented.

Weaknesses

- More detail on the individual components of successful HIV education programs, evidence-based interventions, and the existing HIV gaming literature which will inform the current application would be helpful.
- It is unclear how the gaming content elements of exploring social interactions, such as helping, offending, complimenting, bragging, being humble, competing, empathizing, insulting, challenging, and flirting are derived.

2. Investigator(s):

Strengths

- Mr. Greenberg (MPI) is a gaming expert with extensive experience in successful game applications. He is the Principal Investigator of several SBIRS.
- Dr. Castel (MPI) is a physician-researcher whose expertise is HIV medical epidemiology. She has been the Principal Investigator of several HIV prevention studies and has collaborated with Mr. Greenberg as MPI on two SBIRs.
- Dr. Kuo (Co-investigator) is an infectious disease epidemiologist with extensive HIV research experience.
- Dr. Ciarleglio (Co-investigator) will serve as the biostatistician.
- MPIs have successfully collaborated in the past.
- Dr. D'Angelo (Consultant) is a physician at CNMC and will assist with recruitment.

Weaknesses

- It is unclear who is the behavioral content expert for developing the game scenarios and learning algorithms.

3. Innovation:

Strengths

- Game features include customization of character, choice of partners, choice of forms of sex, personal health risk, and connection to local care. These elements are not present in existing games for HIV education. The "MyLife" game has personalized gameplay that rewards players for HIV testing and linkage to care, and sharing the game with others via social networks.
- The gaming principles are focused on gaming for fun (i.e., entertainment) rather than didactic learning (i.e., a health game).

Weaknesses

- The listed innovations (health promotion, content, commercialization, social media, game construction) are not unique elements in-and-of themselves, but their application to HIV risk games is novel.

4. Approach:

Strengths

- Successful Phase I outcomes support moving forward into a Phase II project.
- Conceptual frameworks to guide intervention development are appropriate and aligned with study goals.
- Expanding the gameplay content should assist with maintaining audience engagement.

- Mixed methods approach to informing the Phase II product.
- Sample size considerations and data analysis for all aims are appropriate.
- RCT study design is clear and logical and the control app is appropriate.
- Overall, the Phase II study strategy is reasonable and seems accomplishable.

Weaknesses

- Application of the CDC Risk Assessment Tool into gaming scenarios and gamer-decisions was not well described.
- For Aim 1, use of a peer referral system for recruitment has potential bias and confidentiality concerns. It is not stated whether known peers will or can participate in focus groups together.
- The standardized survey instruments and IDIs need to be fully described and psychometrics provided.
- In Aim 2, it is unclear what criterion score will be used to confirm acceptability on the measures provided.
- The outcomes of the qualitative data Aim 3 need better description.
- It is unknown whether the research team has had any prior success with using a photo of HIV testing as a confirmation of self-reported testing. Limitations and concerns of this approach were not clearly outlined.
- Generalizability of findings and preferences for gaming content is limited to the DC area.

5. Environment:

Strengths

- Med Rez is a software production company with significant expertise in app and game development.
- George Washington University has excellent resources and access to the target population.
- Strong scientific environment for the proposed work.

Weaknesses

- None noted.

Study Timeline:

Strengths

- Study milestones and timeline are appropriate.

Weaknesses

- The study is 3 years. *(not score driving)*

Phase II (Type 2 R42 and Type 2 R44 applications):

Acceptable

- Preliminary work from Phase I supports moving forward to a Phase II

Direct Phase II (Type 1 R44 applications See the SBIR/STTR Info Form):

Not Applicable

Fast Track (Type 1 R42 and Type 1 R44 applications See the SBIR/STTR Info Form):
Not Applicable

Protections for Human Subjects

Acceptable Risks and Adequate Protections

Data and Safety Monitoring Plan (Applicable for Clinical Trials Only):

Acceptable

- DSMP with Board is proposed.

Inclusion Plans:

- Sex/Gender: Distribution justified scientifically
- Race/Ethnicity: Distribution justified scientifically
- For NIH-Defined Phase III trials, Plans for valid design and analysis: Not applicable
- Inclusion/Exclusion Based on Age: Distribution justified scientifically

Vertebrate Animals:

Not Applicable (No Vertebrate Animals)

Biohazards:

Not Applicable (No Biohazards)

Resubmission:

- Overall, this resubmission is highly responsive to previous reviewers' comments. The weaknesses identified are heavily outweighed by the study's strengths.

Resource Sharing Plans:

Acceptable

Budget and Period of Support:

Recommend as Requested

- Budget is within the 50% above cap limits and has an appropriate budget justification to support.

CRITIQUE 2

Significance: 3
Investigator(s): 1
Innovation: 3
Approach: 3

Environment: 1

Overall Impact: The overall goal of the proposed project is to increase HIV testing among sexually active adolescents and young adults (AYA), aged 13-24. The specific goals of this Phase II are to enhance the existing social media game developed in Phase I through iterative focus testing, pilot test the additions and make revisions, and then test the efficacy of the product via a randomized clinical trial with 300 AYA. HIV screening is low among the AYA population, and if successful, the proposed project could have a significant impact on this important problem. The investigators state that the proposed game improves upon existing products by allowing for extensive personalization, real-time updating of HIV testing locations, and other features not found in current products. Results from the Phase I indicate that all milestones were met, and that users found the game to be engaging and acceptable. The investigators have been responsive to the previous reviewers' concerns, providing additional information and revising methods as per the reviewers' comments. A few concerns remain that somewhat diminish enthusiasm for the proposed project. The commercialization plan is based on the assumption that the app will be monetized through advertising, sponsored content or in-app purchases; however, there is no basis for this assumption given the user population and game content. There is no discussion of the strengths and weaknesses of prior studies nor how the proposed research will overcome the limitations of previous studies. There are several minor weaknesses in the approach. However, on balance, the overall potential of the project is moderately high.

1. Significance:

Strengths

- HIV screening is low among sexually active adolescents and young adults (AYA) aged 13 – 24. This is a significant public health problem which the proposed application aims to address.
- If successful, the proposed product could have a significant positive impact on the health of AYA.
- The proposed game (working title: MyLife) appears to have some additional features and enhancements (e.g., avatar customization, real-time testing locations, more choices within the game, etc.) that may result in increased effectiveness over the existing products on the market.
- The investigators claim that the MyLife game is engaging to users, which is an important element of digital health programs.
- Media Rez is committing monetary resources to product start-up.

Weaknesses

- The investigators do not provide a description of the strengths and weaknesses of prior research in this area, nor do they describe how the proposed project will overcome limitations of previous research to ensure rigor and reproducibility.
- The commercialization plan is based on the assumption that the app will be monetized through advertising, sponsored content or in-app purchases. There is no basis for this assumption given the user population and game content.
- AYA and older adults vs. AYA, respectively). The market is defined as AYA and "older, more affluent players". However, the product is being developed and tested specifically for AYA, not older adults.
- The viral marketing plan is not based on any games with similar content.

2. Investigator(s):

Strengths

- The investigators are extremely well-qualified to carry out the proposed project.
- The Multiple Leadership Plan adequately differentiates the roles of Dr. Greenberg and Dr. Castel and provides a good description of the communication and conflict resolution plans.

Weaknesses

- None noted.

3. Innovation:

Strengths

- Creating a highly customizable, interactive, engaging game to increase HIV testing among AYA is somewhat novel.

Weaknesses

- Gamification of health promotion programs is not novel.
- The commercialization plan is not novel.
- The proposed methods are not novel.

4. Approach:

Strengths

- The methods for focus testing conducted under Aim 1 appear to be appropriate.
- Qualitative and quantitative data will be collected and analyzed during Aim 1 using procedures similar to those used successfully during the Phase I.
- The methods for internal QA/QC and pilot testing of the final game prototype appear to be appropriate and feasible to accomplish.
- The methods for the RCT appear to be appropriate and rigorous, with a clear description of recruitment, enrollment, and randomization procedures.
- The game content will be regularly updated to maintain program engagement.
- Potential pitfalls and alternative approaches are provided.

Weaknesses

- There is a lack of detail regarding how the additional features and modifications identified in Phase I will be operationalized in during the Game Prototype Modifications phase.
- It is not clear how the timeframe for recruitment from previous studies compares to the proposed study, which makes it difficult to assess the feasibility of accrual goals.
- There is a lack of detail regarding final prototype production.
- There is a lack of detail regarding the control condition app to be used in Aim 3.
- The power estimate does not provide an estimate for effect sizes based on prior research and the estimate of 16% attrition seems very low for a digital health study.
- The investigators do not provide information about how they will control for differences in exposure between the “engaging” intervention condition program and the control program.
- There is no discussion of how the possibility of parental consent might affect the investigators’ ability to recruit.

5. Environment:

Strengths

- The environments of Media Rez and George Washington University appear excellent for conducting the proposed project.

Weaknesses

- None noted.

Study Timeline:

Strengths

- Overall, the study timeline appears feasible.

Weaknesses

- The timeline does not provide detail for each activity or clear milestones.
- No alternatives are provided in the Timeline for dealing with potential issues encountered.

Phase II (Type 2 R42 and Type 2 R44 applications):

Acceptable

Direct Phase II (Type 1 R44 applications See the SBIR/STTR Info Form):

Not Applicable

Fast Track (Type 1 R42 and Type 1 R44 applications See the SBIR/STTR Info Form):
Not Applicable

Protections for Human Subjects

Acceptable Risks and Adequate Protections

Data and Safety Monitoring Plan (Applicable for Clinical Trials Only):

Acceptable

Inclusion Plans:

- Sex/Gender: Distribution justified scientifically
- Race/Ethnicity: Distribution justified scientifically
- For NIH-Defined Phase III trials, Plans for valid design and analysis: Not applicable
- Inclusion/Exclusion Based on Age: Distribution justified scientifically

Vertebrate Animals:

Not Applicable (No Vertebrate Animals)

Biohazards:

Not Applicable (No Biohazards)

Resubmission:

- The investigators have been fairly responsive to the previous reviewers' concerns, highlighting their contributions to science and commercialization of products developed in previous SBIR projects, clarifying differences in the proposed product from existing products, providing greater detail in the Approach section, and making revisions to methods as per the reviewers' suggestions.
- Although the investigators have clarified details in their commercialization plan, the assumptions upon which the plan is based do not necessarily apply to the topic and population targeted by the game, and there are discrepancies in the description of the market.

Resource Sharing Plans:

Acceptable

Budget and Period of Support:

Recommend as Requested

CRITIQUE 3

Significance: 3
Investigator(s): 1
Innovation: 3
Approach: 2
Environment: 1

Overall Impact: This proposed Phase II SBIR project seeks to promote HIV testing among adolescents and young adults (AYA) through an avatar-based life simulation game with educational content related sexual health decision-making. The investigators have been responsive to the prior review. The application addresses a problem of high significance, and the investigators and environment are very strong. Given the long development and evaluation timeframe compared to the fast pace of commercial game development and given the number of other games on the market vying for AYA's attention there is a concern that the game may have limited uptake.

1. Significance:

Strengths

- AYA account for a notable proportion of new HIV infections and many AYA with HIV do not know they're infected.
- Existing strategies to increasing HIV testing in AYA have met with limited success. An engaging game could perhaps reach a significant proportion of AYA who have as yet not been engaged.
- The rigor of the prior research is strong.

Weaknesses

- Though focus group feedback seemed promising, it is difficult to gauge the potential uptake of such a game when there are so many other kinds of games available for AYA.

2. Investigator(s):

Strengths

- The Principal Investigators have collaborated since 2014.
- Principal Investigator Greenberg has one completed Phase II SBIR and previously created games that met with commercial success
- Principal Investigator Castel is a pediatrician and HIV epidemiologist with significant experience conducting research.
- Co-investigators bring expertise in infectious disease and biostatistics.

Weaknesses

- None noted.

3. Innovation:

Strengths

- This resubmission clarifies the innovation of the of the proposed game. It seeks to shift practice by creating a game for physicians, schools, etc., to give to AYA to educate them about HIV, which would be novel. Allowing users freedom to choose their sex/gender, sex partners' sex/gender, forms of sex, etc., is also innovative.

Weaknesses

- Educational avatar-based life simulation games have been used in other contexts.

4. Approach:

Strengths

- Phase I focus group testing indicated high acceptability in providers and AYA.
- The Phase II development plan is strong and incorporated formative evaluation in the form of focus group testing and pilot testing to determine usability. Methodologies are transparent and appropriate.
- Summative evaluation will be an efficacy trial with an active control condition (existing HIV educational material). The study is designed with good scientific rigor. Experimental controls will be implemented to enhance internal validity. Potential confounders are addressed.
- The power analysis and data analysis plan are appropriate.
- Potential pitfalls and alternative approaches are considered.
- The resubmission includes a revised plan to stratify the focus groups by age and to obtain parental consent should a waiver of consent be unavailable.

Weaknesses

- The research plan is highly ambitious, with the project requiring 3 years. Given the fast pace at which new and innovative games are released, this risks a situation where the game is already a bit dated by the time it is commercialized.

5. Environment:

Strengths

- The facilities and other resources of Media Rez and GWU are more than adequate to conduct the proposed research.

Weaknesses

- None noted.

Study Timeline:

Strengths

- The timeline is long but reasonable given the aims.
- The timeline has well-specified milestones.

Weaknesses

- None noted.

Phase II (Type 2 R42 and Type 2 R44 applications):

Acceptable

- The Phase I project was successful and the commercialization plan is strong.

Direct Phase II (Type 1 R44 applications See the SBIR/STTR Info Form):

Not Applicable

Fast Track (Type 1 R42 and Type 1 R44 applications See the SBIR/STTR Info Form):
Not Applicable

Protections for Human Subjects

Acceptable Risks and Adequate Protections

- The investigators have attended to the risks involved in a study regarding sexual behavior and HIV in minors.

Data and Safety Monitoring Plan (Applicable for Clinical Trials Only):

Acceptable

- A DSMB will be used. Plans are appropriate.

Inclusion Plans:

- Sex/Gender: Distribution justified scientifically
- Race/Ethnicity: Distribution justified scientifically
- For NIH-Defined Phase III trials, Plans for valid design and analysis: Not applicable
- Inclusion/Exclusion Based on Age: Distribution justified scientifically
- Children and adults will be included. Gender will be equally distributed and minorities will be appropriately represented.

Vertebrate Animals:

Not Applicable (No Vertebrate Animals)

Biohazards:

Not Applicable (No Biohazards)

Resubmission:

- The investigators have been thoroughly responsive to the prior critiques and made meaningful changes to the research plan.

Resource Sharing Plans:

Not Applicable (No Relevant Resources)

Budget and Period of Support:

Recommend as Requested

THE FOLLOWING SECTIONS WERE PREPARED BY THE SCIENTIFIC REVIEW OFFICER TO SUMMARIZE THE OUTCOME OF DISCUSSIONS OF THE REVIEW COMMITTEE, OR REVIEWERS' WRITTEN CRITIQUES, ON THE FOLLOWING ISSUES:

PROTECTION OF HUMAN SUBJECTS: ACCEPTABLE

INCLUSION OF WOMEN PLAN: ACCEPTABLE

INCLUSION OF MINORITIES PLAN: ACCEPTABLE

INCLUSION ACROSS THE LIFESPAN PLAN: ACCEPTABLE

COMMITTEE BUDGET RECOMMENDATIONS: The budget was recommended as requested.

Footnotes for 2 R44 HD088332-02A1; PI Name: Greenberg, Daniel J

NIH has modified its policy regarding the receipt of resubmissions (amended applications). See Guide Notice NOT-OD-14-074 at http://grants.nih.gov/grants/guide/notice-files/NOT-OD-14-074.html. The impact/priority score is calculated after discussion of an application by averaging the overall scores (1-9) given by all voting reviewers on the committee and multiplying by 10. The criterion scores are submitted prior to the meeting by the individual reviewers assigned to an application, and are not discussed specifically at the review meeting or calculated into the overall impact score. Some applications also receive a percentile ranking. For details on the review process, see http://grants.nih.gov/grants/peer_review_process.htm#scoring.

MEETING ROSTER

Center for Scientific Review Special Emphasis Panel
CENTER FOR SCIENTIFIC REVIEW
Small Business: Disease Prevention and Management, Risk Reduction and Health Behavior Change

ZRG1 RPHB-Z (10)
06/24/2019 - 06/25/2019

Notice of NIH Policy to All Applicants: Meeting rosters are provided for information purposes only. Applicant investigators and institutional officials must not communicate directly with study section members about an application before or after the review. Failure to observe this policy will create a serious breach of integrity in the peer review process, and may lead to actions outlined in NOT-OD-14-073 at https://grants.nih.gov/grants/guide/notice-files/NOT-OD-14-073.html and NOT-OD-15-106 at https://grants.nih.gov/grants/guide/notice-files/NOT-OD-15-106.html, including removal of the application from immediate review.

CHAIRPERSON(S)

POLLIO, DAVID E, PHD
DISTINGUISHED PROFESSOR AND CHAIR
DEPARTMENT OF SOCIAL WORK
THE UNIVERSITY OF ALABAMA AT BIRMINGHAM
BIRMINGHAM, AL 35294

MEMBERS

ARCOLEO, KIMBERLY JOAN, PHD, MPH
PRINCIPAL INVESTIGATOR
ABIGAIL WEXNER RESEARCH INSTITUTE AT NATIONWIDE
CHILDREN'S HOSPITAL
OHIO STATE UNIVERSITY
ROCHESTER, NY 14642

BESSESEN, DANIEL HOLLAND, MD
PROFESSOR
DIVISION OF ENDOCRINOLOGY AND METABOLISM
DENVER HEALTH MEDICAL CENTER
UNIVERSITY OF COLORADO
DENVER, CO 80204

BOHNERT, AMY S.B., PHD
ASSOCIATE PROFESSOR
DEPARTMENT OF PSYCHIATRY
UNIVERSITY OF MICHIGAN
ANN ARBOR, MI 48109

BUSINELLE, MICHAEL S, PHD
ASSOCIATE PROFESSOR/ CO-DIRECTOR/ DIRECTOR
OKLAHOMA TOBACCO RESEARCH CENTER
UNIVERSITY OF OKLAHOMA HEALTH SCIENCES CENTER
M HEALTH RESOURCES
STEPHSON CANCER CENTER
OKLAHOMA CITY, OK 73104

DEWAN, MAYA L, MD
ASSISTANT PROFESSOR,
DIVISION OF CRITICAL CARE MEDICINE
UC DEPARTMENT OF PEDIATRICS
CINCINNATI CHILDREN'S
CINCINNATI, OH 45229

DUNN, KELLY E., PHD
ASSOCIATE PROFESSOR
DEPARTMENT OF PSYCHIATRY AND BEHAVIORAL
SCIENCES
SCHOOL OF MEDICINE
JOHNS HOPKINS UNIVERSITY
BALTIMORE, MD 21224-6823

GORDON, JUDITH S, PHD
PROFESSOR
COLLEGE OF NURSING AND DEPARTMENT OF FAMILY
AND COMMUNITY MEDICINE
UNIVERSITY OF ARIZONA
TUCSON, AZ 85721

HAAS, NIINA M., MA
VICE PRESIDENT
BRIGHT OUTCOME INC.
BUFFALO GROVE, IL 60089-1998

HARRINGTON, CHERISE BALDWIN, MPH, PHD
ASSOCIATE PROFESSOR
DEPARTMENT OF PUBLIC HEALTH EDUCATION
NORTH CAROLINA CENTRAL UNIVERSITY
DURHAM, NC 27707

HERMAN, CARLA JEAN, MD
PROFESSOR
DEPARTMENT OF INTERNAL MEDICINE
SCHOOL OF MEDICINE ALBUQUERQUE, NM
UNIVERSITY OF NEW MEXICO
ALBUQUERQUE, NM 87106

HICKAM, DAVID H, MD
DIRECTOR OF CLINICAL EFFECTIVENESS PROGRAM
DIRECTOR OF CLINICAL EFFECTIVENESS PROGRAM
PATIENT CENTERED OUTCOMES RESEARCH INSTITUTE
WASHINGTON, DC 20036

HINGLE, MELANIE DANIELA, PHD
ASSOCIATE PROFESSOR
DEPARTMENT OF NUTRITIONAL SCIENCES
COLLEGE OF AGRICULTURE AND LIFE SCIENCES
THE UNIVERSITY OF ARIZONA
TUCSON, AZ 85721

JOHNSON, ROBIN, PHD
CONSULTANT
OXFORD CONSULTANTS
BEND, OR 97701

KALARCHIAN, MELISSA A, PHD
PROFESSOR OF NURSING AND PSYCHOLOGY / ASSOCIATE DEAN FOR RESEARCH, SCHOOL OF NURSING
DUQUESNE UNIVERSITY
PITTSBURGH, PA 15232

KIM, TAE YOUN, PHD
ASSOCIATE PROFESSOR
SCHOOL OF NURSING
UNIVERSITY OF CALIFORNIA, DAVIS
SACRAMENTO, CA 95817

LYONS, ELIZABETH J., PHD
ASSOCIATE PROFESSOR
DEPARTMENT OF NUTRITION AND METABOLISM
THE UNIVERSITY OF TEXAS MEDICAL BRANCH
GALVESTON, TX 77550

MANSKI, RICHARD J, PHD
PROFESSOR AND CHAIR
DEPARTMENT OF DENTAL PUBLIC HEALTH
UNIVERSITY OF MARYLAND SCHOOL OF DENTISTRY
BALTIMORE, MD 21201

MCQUAID, ELIZABETH L., PHD
PROFESSOR
DEPARTMENT OF PSYCHIATRY & HUMAN BEHAVIOR
RHODE ISLAND HOSPITAL
BROWN MEDICAL SCHOOL
BROWN UNIVERSITY
PROVIDENCE, RI 02903

MILLER, CARLA K, PHD
PROFESSOR
DEPARTMENT OF HUMAN SCIENCES
OHIO STATE UNIVERSITY
COLUMBUS, OH 43210

MYERS, VALERIE H, PHD
SENIOR SCIENTIST
HEALTH COMMUNICATION RESEARCH
AND MULTIMEDIA DEVELOPMENT
KLEIN BUENDEL, INC
GOLDEN, CO 80401

NAKAMURA, YOSHIO, PHD
ASSOCIATE PROFESSOR
DEPARTMENT OF ANESTHESIOLOGY
PAIN RESEARCH CENTER
UNIVERSITY OF UTAH
SALT LAKE CITY , UT 84108

NELSON, SARAH E, PHD
ASSOCIATE DIRECTOR OF RESEARCH
DIVISION ON ADDICTION
CAMBRIDGE HEALTH ALLIANCE
HARVARD MEDICAL SCHOOL
MEDFORD, MA 02155

OSBORN, CHANDRA Y, PHD
CHIEF BEHAVIORAL OFFICER
LIRIO
KNOXVILLE, TN 37923

RAPCHAK, BARBARA ANN, BS
VICE PRESIDENT
VICE PRESIDENT RESEARCH & DEVELOPMENT
INTELLIGENT MEDICAL OBJECTS, INC
NORTHBROOK, IL 60062

RICHARDS, ERIC A, PHD
CHIEF EXECUTIVE OFFICER
STEPPING STONES, INC
HUNTSVILLE, AL 35805

SACCONE, NANCY L, PHD
ASSOCIATE PROFESSOR
DEPARTMENT OF GENETICS
WASHINGTON UNIVERSITY SCHOOL OF MEDICINE
ST. LOUIS, MO 63110

SHEINFELD GORIN, SHERRI, PHD
PROFESSOR OF RESEARCH AND DIRECTOR
NEW YORK PHYSICIANS AGAINST CANCER
NEW YORK CITY, NY 11733

STERNS, ANTHONY ALEXANDER, PHD
CEO
IRXREMINDER LLC
CLEVELAND, OH 44114

STONER, SUSAN A, PHD
RESEARCH CONSULTANT
ALCOHOL AND DRUG ABUSE INSTITUTE
UNIVERSITY OF WASHINGTON
SEATTLE, WA 98105

TSOW, FRANCIS, PHD
RESEARCH ASSISTANT PROFESSOR
DEPARTMENT OF BIOMEDICAL ENGINEERING
VANDERBILT UNIVERSITY
NASHVILLE, TN 37240

WALDRON, HOLLY BARRETT, PHD
RESEARCH SCIENTIST
OREGON RESEARCH INSTITUTE
EUGENE, OR 97403

SCIENTIFIC REVIEW OFFICER

MCQUESTION, MICHAEL J, PHD
SCIENTIFIC REVIEW OFFICER
CENTER FOR SCIENTIFIC REVIEW
NATIONAL INSTITUTES OF HEALTH
BETHESDA, MD 20892

EXTRAMURAL SUPPORT ASSISTANT

NJUKI, JENNIFER N, BS
EXTRAMURAL SUPPORT ASSISTANT
CENTER FOR SCIENTIFIC REVIEW
NATIONAL INSTITUTES OF HEALTH
BETHESDA 20892

Consultants are required to absent themselves from the room during the review of any application if their presence would constitute or appear to constitute a conflict of interest.
